# The power of partnerships: state public health department multisector collaborations in major chronic disease programme areas in the United States

**DOI:** 10.1186/s12961-021-00765-3

**Published:** 2022-07-08

**Authors:** Edward Tsai, Peg Allen, Louise Farah Saliba, Ross C. Brownson

**Affiliations:** 1 Division of Public Health Sciences, Department of Surgery, and Alvin J. Siteman Cancer Center, Washington University School of Medicine, Washington University in St. Louis, 660 S. Euclid Avenue, Campus Box 8100, St. Louis, MO 63110 USA; 2 Prevention Research Center, Brown School at Washington University in St. Louis, St. Louis, USA

**Keywords:** Public health, State health department, Chronic disease, Multisector collaboration, Health equity

## Abstract

**Background:**

Multisector collaboration between state public health departments (SHDs) and diverse community partners is increasingly recognized as important for promoting positive public health outcomes, addressing social determinants of health, and reducing health inequalities. This study investigates collaborations between SHDs in the United States and different types of organizations addressing chronic disease in and outside of the health sector.

**Methods:**

SHD employees were randomly selected from the National Association of Chronic Disease Directors membership list for participation in an online survey. Participants were asked about their primary chronic disease work unit (cancer, obesity, tobacco, diabetes, cardiovascular disease, and others), as well as their work unit collaborations (exchange of information/cooperation in activities) with organizations in health and non-health sectors. As a measure of the different organizations SHDs collaborated with in health and non-health sectors, a collaboration heterogeneity score for each programme area was calculated. One-way analysis of variance (ANOVA) with Tukey’s post hoc tests were used to assess differences in collaborator heterogeneity between programme areas.

**Results:**

A total of 574 participants were surveyed. Results indicated that the cancer programme area, along with diabetes and cardiovascular disease, had significantly less collaboration heterogeneity with organizations outside of the health sector compared to the obesity and tobacco programme areas.

**Conclusions:**

While collaborations with health sector organizations are commonly reported, public health departments can increase collaboration with sectors outside of health to more fully address chronic disease prevention.

**Supplementary Information:**

The online version contains supplementary material available at 10.1186/s12961-021-00765-3.

## Background

Multisector interagency collaboration is necessary to implement the complex multilevel evidence-based health policies, environmental interventions, and systems approaches that address social determinants of health for the major chronic diseases that have become a core focus for public health [1, 2]. In recognition of this, policy actions such as the Health in All Policies initiatives launched in Europe in the early 2000s have since been adopted in many countries to bring diverse sectors such as transportation, land use, housing, and education together to improve population health and health equity by incorporating health considerations into policy-making [3–8]. Multisectoral collaboration is essential to reducing poverty, improving education opportunities, creating equitable built environments, and meeting key United Nations (UN) Sustainable Development Goals to improve well-being for all [9–11]. Governmental public health departments have a critical role to play in these initiatives and have increasingly collaborated with other health organizations such as clinics and hospitals, with calls for increased partnerships with a broader array of organizations [12]. In the United States, each of the 50 states has primary authority to address and protect the public’s health under its constitutional doctrine of reserved powers [13]. Public health practitioners in United States state health departments (SHDs) can develop and ensure effective implementation strategies for delivery of evidence-based programmes and policies to address public health problems [14]. In the United States, chronic disease funding comes largely from federal agencies to SHDs, which then contract with local agencies such as local health departments (LHDs) for implementation of evidence-based interventions the SHD approves. Many of the evidence-based interventions, such as those to reduce tobacco use or increase physical activity opportunities, are multilevel and involve complex system-wide and/or environmental and policy changes [15]. Such changes involve collaboration with organizations in sectors within and outside of health [1, 2, 15].

In recent decades, the United States Department of Health and Human Services (the federal-level executive branch department responsible for services related to health) through the Healthy People objectives, along with the Institute of Medicine (now the National Academy of Medicine), have continuously advocated for stronger collaborations between public health departments and multisector organizations [16]. For example, core Healthy People agenda items starting from the turn of the century have called for SHDs to take a leadership role in collaborating with diverse partners to facilitate the implementation of community health improvement plans [17]. In the same time frame, Institute of Medicine recommendations from annual reports have called for collaborative partnerships as a key mechanism for involving organizations with a stake in community health [18].

Despite this, the extent of widespread public health department collaboration with multisector organizations remains unclear, with some evidence suggesting multisector partnerships are beneficial for community health, but that public health departments still collaborate primarily with organizations within the health sector [17, 19, 20]. Participants in Canada’s Multi-sectoral Partnerships Initiative in public health reported increased resources, including increased access to people with different skills and expertise [21, 22]. Additionally, most studies in the area of public health collaborations have focused primarily on characterizing smaller-scale partnerships between local-level health departments and agencies [18]. Less is known on how mid- to higher-level public health agencies such as SHDs collaborate with organizations outside of the health sector. Evidence at the local level shows benefits of multisector community partnerships. For example, a national study by Tabak et al. (2018) found that for LHDs in the United States, collaboration with other multisector organizations in the community was critical for the provision of evidence-based interventions related to obesity and diabetes prevention, as few interventions were delivered directly by the LHD itself [23]. In the area of cardiovascular disease, a local multisector initiative resulted in increased percentages of healthcare system hypertensive patients with controlled blood pressure, compared to baseline [24]. Multisector cancer collaborations have shown increased use of evidence-based approaches to facilitate cancer screening [25, 26] and increased cancer screening rates [27]. Although hospitals are required to work with community-based organizations to inform their community health needs assessments and plans, such collaborations are often not sustained through implementation [28].

While evidence in local-level partnerships is promising, key differences exist in the roles and collaborative and partnership-forming processes of state-level health departments versus local-level health agencies. For example, compared to LHDs, SHDs are expected to take on a more central leadership role, to be involved in higher-level activities such as informing statewide health policy creation, and to manage relationships with diverse partner organizations and many different LHDs that may have competing priorities [18, 29].

Additionally, SHDs increasingly focus on health equity at a systems level and can serve as a bridging hub to foster both state- and local-level multisector collaborations to address health equity and social determinants of health. For example, poverty is linked to higher morbidity and mortality from cancer and other chronic diseases [30, 31]. Inadequate housing and neighbourhood air pollution are associated with emergency room visits for asthma [32–36]. Transportation barriers are associated with missed preventive screenings, delayed cancer diagnoses, and inadequate management of chronic diseases [37–41]. Food insecurity and inadequate access to healthy foods are associated with higher rates of obesity and other cardiometabolic conditions [42–44] and possibly lung function [45]. Although evidence is still developing, there is a substantial body of research linking social determinants of health to a range of health outcomes [46]. More work is needed on which actions taken by sectors outside of the health sector will most effectively improve social determinants of health [2, 47]. Because social determinants of health are impacted by a diverse array of service sectors outside of health-focused organizations like hospitals and clinics (e.g., housing, transportation, schools, city planning), SHDs are often in a position to facilitate partnerships between organizations belonging to different sectors [12]. In the context of community health equity, evidence suggests that in state-level public health practice, a positive association exists between higher-quality, more diverse partnerships and commitment to health equity work through efforts to reduce poverty and social needs among population groups experiencing excess disease burden [48]. A recent systematic review on public health strategies to reduce health inequalities additionally identified multisector collaborations by public health agencies as a core component for successful interventions and programmes [49]. The Consolidated Framework for Collaboration Research posits that multisector collaborations with broad representation across sectors, breadth of active memberships, and community representation, in combination with leadership and workgroup best practices, can leverage social capital and group dynamics to build capacity and increase community engagement and creditability of collaborations, ultimately leading to the desired public health outcomes of reduced health disparities and improved equity [47]. As many of the social determinants of health that lead to health inequalities (e.g., unequal access to education and housing) lie outside of the domain of the healthcare sector, the Consolidated Framework for Collaboration Research describes pathways through which public health collaborations with multisector organizations can help facilitate increased health equity [47].

The Association of State and Territorial Health Officials (ASTHO) report on multisector collaboration among state health agencies provides the best picture of United States state-level health agency collaboration in the literature [19]. This is a valuable and critical source of information on health agency collaborations at the state level, but the ASTHO survey assesses collaboration with a broad lens and does not break down multisector collaborations by chronic disease programme area or work unit within each SHD. This is a gap, as SHDs are not monolithic entities and may contain numerous programme area work units focused on different infectious and chronic diseases. Depending on the specific chronic disease area, these different work units will be engaged in different types of health-promoting activities, thereby necessitating different types of organizational partners and collaborators. For example, a public health work unit focused on reducing childhood obesity may be more likely to collaborate with the parks and recreation department (to promote outdoor physical activity) compared to a work unit in the tobacco control programme area.

This paper aims to provide a snapshot of the types of organizations that SHDs in the United States collaborate with for chronic disease prevention, the degree to which collaborating organizations lie in the health sector versus other sectors, and whether/how collaborations differ depending on specific chronic disease programme areas. Therefore, this study adds to the growing body of literature on multisector collaboration by focusing on state/province-level public health department partnerships and characterizing collaborations within specific chronic disease programme areas.

## Methods

The survey used was part of a larger study investigating factors associated with mis-implementation of public health programmes in SHDs. The study protocol for this larger overall project was reported previously [50]. Participants for the survey were SHD employees recruited from the National Association of Chronic Disease Directors (NACDD) membership list working in SHD chronic disease units. The NACDD is a professional association composed of United States public health workforce members including all SHD chronic disease directors along with SHD staff members. Participants were selected using a random number generator from the NACDD membership list after individuals from territories and non-qualifying positions (e.g., clerical and financial personnel) were excluded. Emails were sent out in June 2018 inviting a random sample of 1239 members (of 3117) to participate in a Qualtrics online survey that remained open for participation until August 2018. Participants were offered a $20 Amazon gift card or to have a donation made to a public health charity of their choosing. Human subjects approval was obtained from the Washington University in St. Louis Institutional Review Board (#201812062).

### Measures

The survey included items on respondent demographics, chronic disease programme working area, experience, and training, which were used in a separate study investigating factors associated with mis-implementation of chronic disease public health programmes in SHDs (Padek et al. 2021). The final survey draft underwent cognitive response testing with 11 former SHD chronic disease directors. Reliability test–retest of the revised draft with 39 current SHD chronic disease unit staff found consistency in scores, and only minor changes to the survey were needed.

The types of organizations that SHDs collaborated with were assessed by providing a roster of possible organization types with the prompt “Which types of organizations does your work unit currently collaborate with?” For each potential partner organization type, respondents were given the option to select any of “exchange information”, “work together on activities or projects”, “my agency provides financial resources”, “my agency serves in a leadership role in the collaboration”, and “my agency is a recipient of financial resources from this organization” to indicate the specific nature of collaboration. Two sets of rosters were provided: one with a list of potential collaborating organizations in the health sector (LHDs, other SHDs, Federally Qualified Health Centers [FQHCs] which are federally designated outpatient clinics receiving funding to serve low-income clients, hospitals, universities/schools/departments focused on health, Indian Health Service, tribal health organizations, Medicaid unit of state agency, state medical associations representing the physician professional society of the state, health nonprofits, health insurance providers, mental health services, foundation/public health institutes), and another with potential collaborators in non-health sectors (universities/schools/departments non-health-focused, primary and secondary school-age education/youth programmes, media/communications/public relations organizations, community development organizations, social services other than Medicaid, businesses, parks and recreation departments, housing, city planning/transportation agencies, justice system, state commissions/special counsels, tribal agencies, other state agencies).

### Analysis

Analysis was conducted on the following chronic disease programme areas: cancer prevention and control, obesity, tobacco, diabetes, and cardiovascular disease. A sixth category was included titled “multiple programme areas”, which was the option respondents selected if they worked in more than one programme area, as many SHD programme managers and section leaders do. Based on the two survey rosters provided, all possible collaborating organization types were categorized into one of two sectors, either “health sector” (including organizations providing healthcare-related services) or “multisector” (including organizations not directly providing health services or not in healthcare-related fields). Collaboration was considered to exist if a respondent indicated that they exchanged information and/or worked together on activities or projects with a particular organization.

**Fig. 1:**
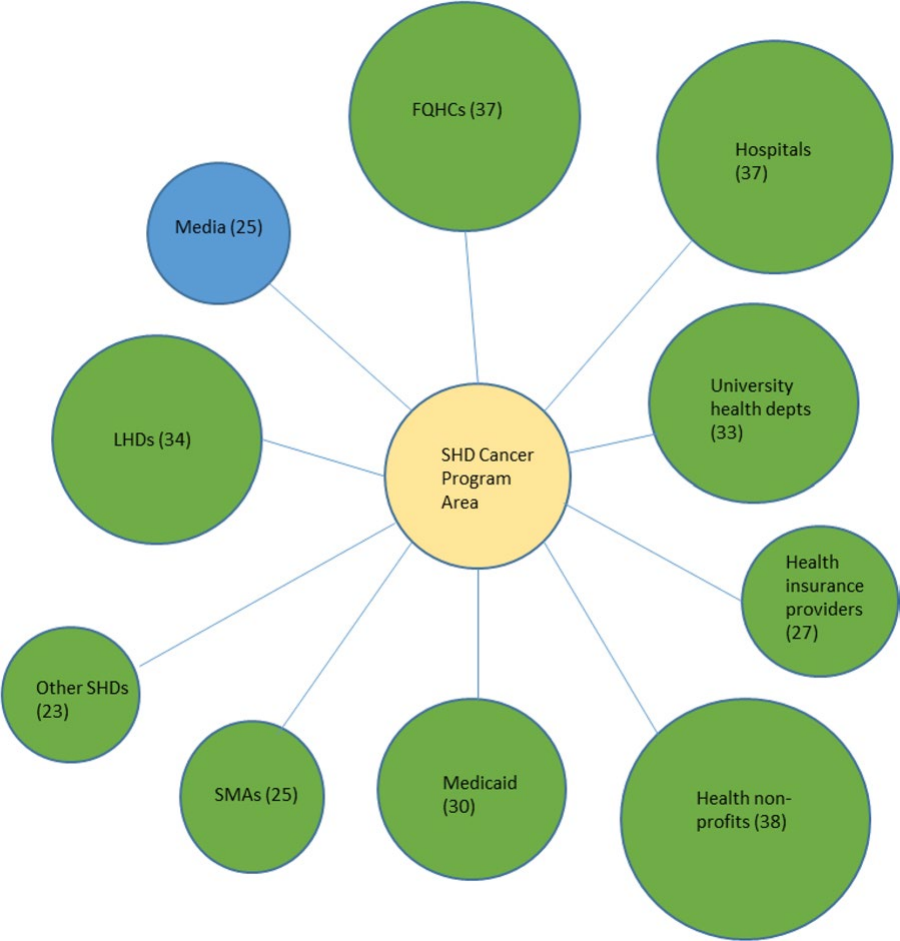
Top 10 SHD collaborators in the cancer programme area. LHD, local health department; SMA, state medical association; FQHC, Federally Qualified Health Center

**Fig. 2:**
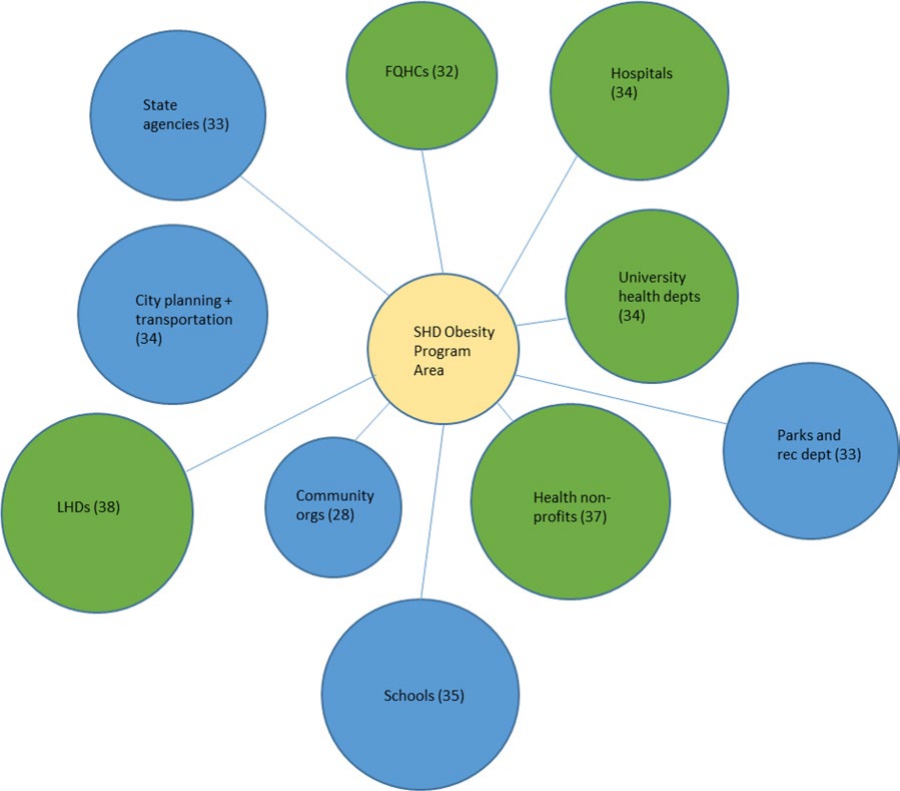
Top 10 SHD collaborators in the obesity programme area

**Fig. 3:**
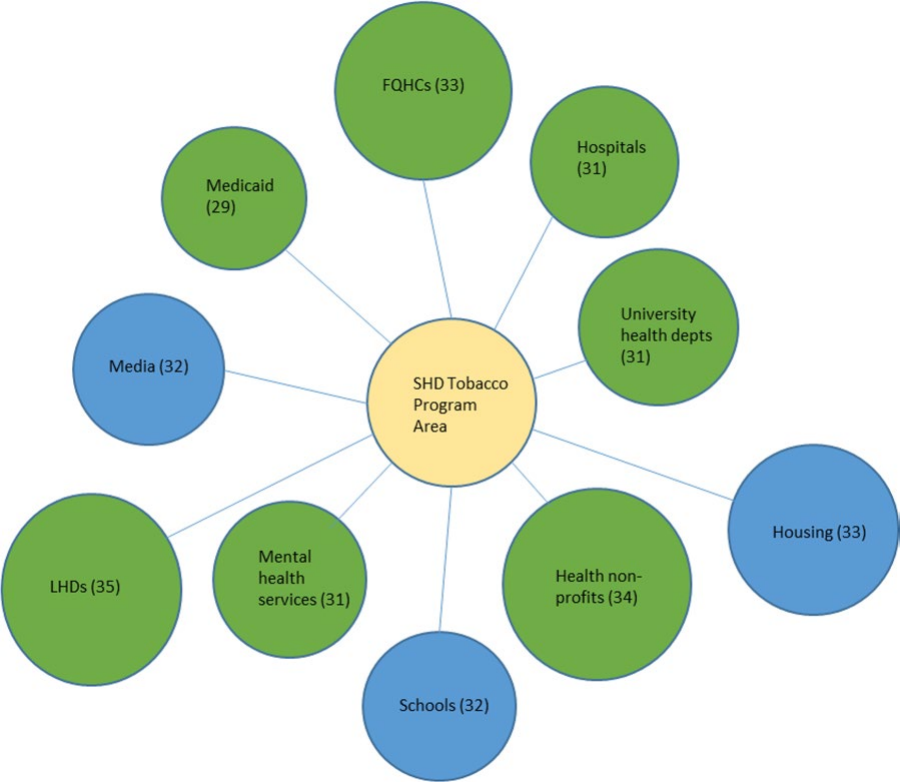
Top 10 SHD collaborators in the tobacco programme area

**Fig. 4:**
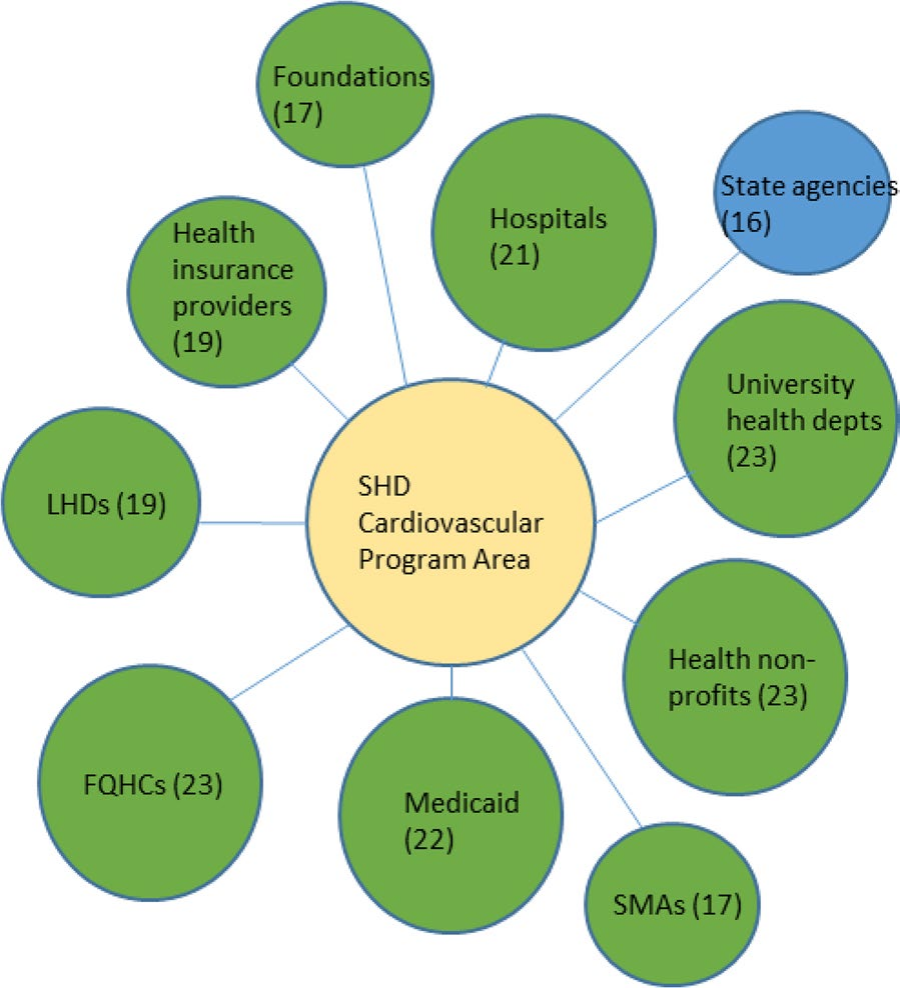
Top 10 SHD collaborators in the cardiovascular programme area

**Fig. 5:**
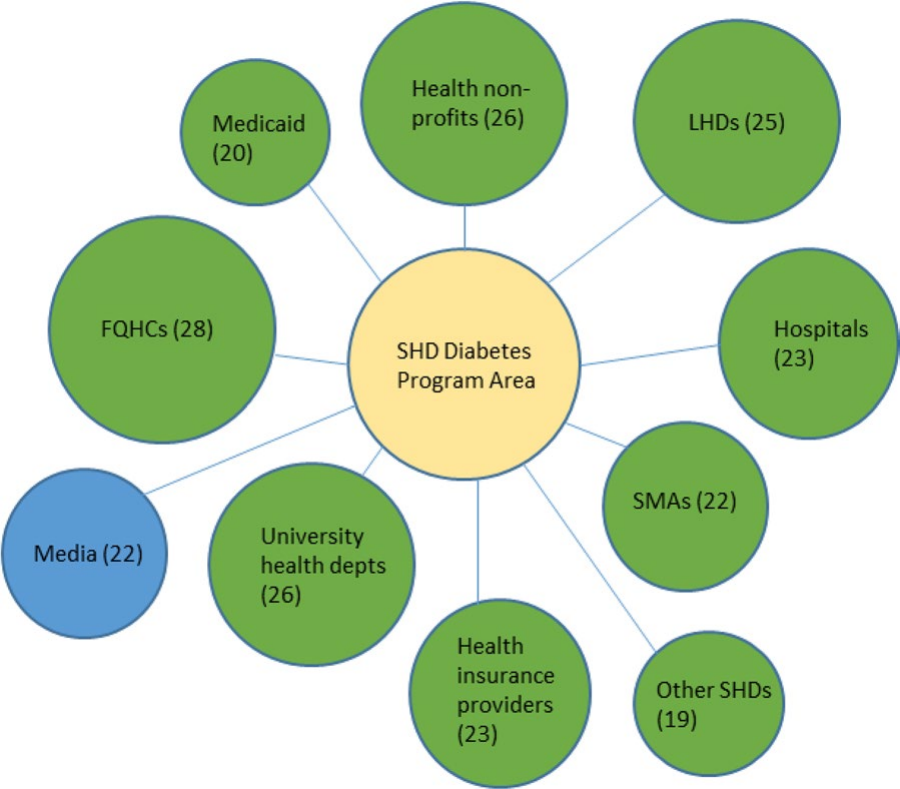
Top 10 SHD collaborators in the diabetes programme area

**Fig. 6:**
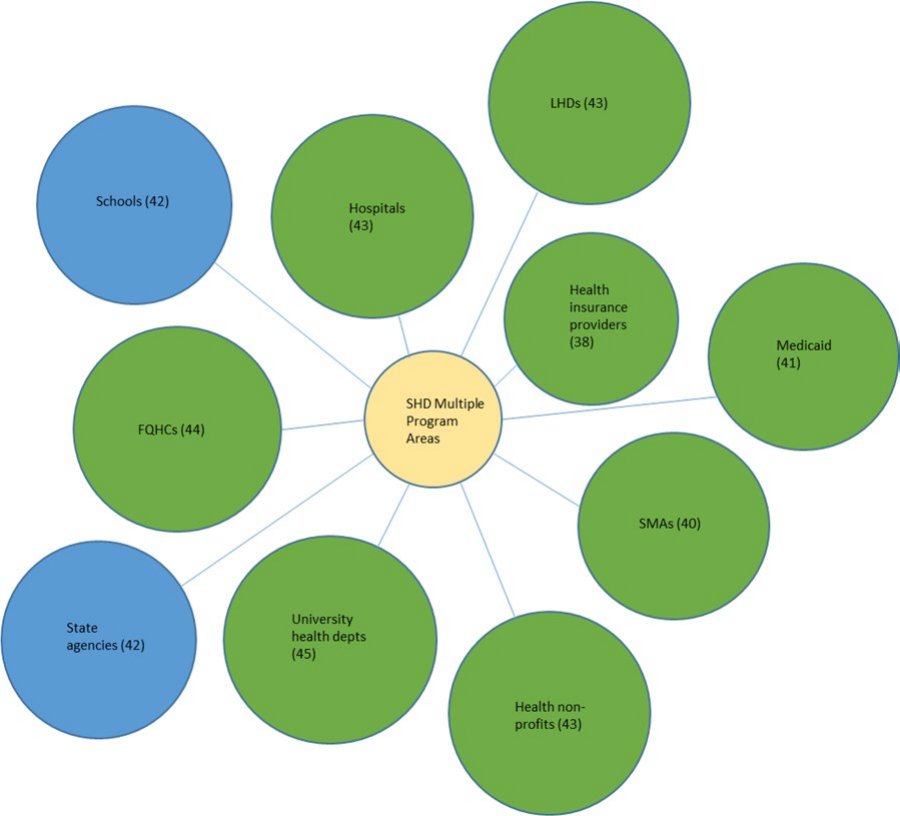
Top 10 SHD collaborators in the “working in multiple chronic disease programme areas”

Collaboration survey responses were combined for each SHD chronic disease programme area so that in cases where there was more than one respondent for any particular programme area in a given state, their responses for organizations collaborated with were aggregated into a single case for that state. Table 1 contains the initial total collected sample size for each chronic disease programme area, as well as the aggregated sample size used for analysis in Figs. 1, 2, 3, 4, 5, 6, and Additional file 1: all appendix figures. As a measure for heterogeneity of organizations collaborated with in the health sector versus multisector, the probability that any two randomly selected organizations collaborating with a SHD in a programme area would be in different sectors was calculated, normalized to a 0–1 scale. Therefore, the higher this heterogeneity number (i.e., the closer the value to 1), the greater the likelihood that any two randomly selected collaborators for a SHD programme area would be from different sectors, indicating greater heterogeneity (i.e., multisector collaborations) in the types of organizations that SHD programme area is working with. This measure, known as the Agresti index of qualitative variation, is a commonly used statistic in fields including sociology, biology, and public health to indicate heterogeneity/diversity with respect to any particular characteristic among a group of entities, for example, gender heterogeneity among a group of students, species diversity in a habitat, and in this case, the heterogeneity of organizational sector types that SHDs collaborate with [51, 52]. Analysis of variance (ANOVA) with Tukey’s honestly significant difference (HSD) post hoc test was conducted to assess potential significant differences in heterogeneity of collaborators across the six chronic disease programme areas. Analysis was conducted and figures were created using SPSS 26.

**Table 1:** Interorganizational collaboration scores by programme area in a 2018 survey of SHD health promotion/chronic disease units, United States

Programme area	Total number of respondents	*N*^a^	Collaborator heterogeneity score^b^ (± SD)
Cancer prevention and control	78	39	0.82 (0.24)
Obesity prevention and management, including physical activity, healthy eating, and obesity screening and management	75	40	0.97 (0.05)
Tobacco use prevention and control	61	36	0.93 (0.09)
Diabetes prevention and management	34	29	0.82 (0.10)
Cardiovascular disease and stroke prevention	42	24	0.78 (0.21)
Multiple health promotion/noncommunicable disease programme areas	156	46	0.82 (0.13)

^a^This *N* represents the final number of SHDs in each chronic disease programme area out of a total possible maximum of 50 states. In cases of multiple respondents in a programme area for a state, these were aggregated into one case

^b^Score indicates probability (on a scale of 0 to 1) that any two randomly selected organization types collaborating with a SHD will be in different sectors. Therefore, the closer the value is to 1, the greater the heterogeneity a SHD will have among collaborating organization types

## Results

The response rate of the overall larger survey was 48.3% (*n* = 643), with some additional dropout for the collaboration portion of the survey 43.1% (*n* = 574). There were respondents from every state in the United States, but not all chronic disease area work units were represented for every state. The number of respondents for the six programme areas of focus is shown in Table 1. Regarding the distribution of participation across ASTHO-defined regions (i.e., New England, South, West, Mountains/Midwest, Mid-Atlantic/Great Lakes), there was relatively even distribution (within a range of 16.5% of total respondents in the least represented region to 23.3% in the most represented region). Most participants were programme managers (50.7%) or specialists in specific roles such as epidemiologist or health educator (33.0%). Further information on participant demographics, background education and training, and evidence-based decision-making capacity have been reported previously [53].

We created diagrams depicting the top 10 types of organizations SHDs collaborated with the most (regardless of sector) for each of the six SHD programme areas of focus (see Figs. 1, 2, 3, 4, 5, 6). Node sizes in the diagram reflect the number of collaborations SHD chronic programme areas have with organizations in and outside of the health sector. Thus, larger-sized organization type nodes indicate that a greater number of SHDs reported collaborating with a particular organization type, compared to a smaller-sized node of another organization type. Blue circles signify an organization type within the health sector. Green circles indicate organization types from sectors outside of health. From Figs. 1, 2, 3, 4, 5, and 6, the obesity and tobacco programme areas collaborated the most with organizations outside of the health sector, with 50% (5/10) and 30% (3/10), respectively, of the organization types that obesity and tobacco collaborated with coming from outside of the health sector. In the cancer, cardiovascular disease, and diabetes SHD programme areas, 10% (1/10) of SHDs most frequently collaborated with organizations coming from outside of the health sector. Additional figures showing the collaboration frequency of all 26 organization types for both the health and multisector can be found in Additional file 1: Appendix A. As shown in Additional file 1: Appendix A, few SHD participants reported collaborations with sectors in business, social services, housing, city planning, or the criminal justice system.

These findings are supported by the collaborator heterogeneity measure shown in Table 1. If any two organization types are selected at random, the obesity (0.97 ± 0.05) and tobacco (0.93 ± 0.09) SHD programme areas have the highest probability of the two selected organizations being from different sectors, therefore indicating that these two programmes possess a greater tendency for heterogeneity among their collaborators. Cancer (0.82 ± 0.24), cardiovascular (0.78 ± 0.21), and diabetes (0.82 ± 0.10) programme areas have the lowest probabilities of collaborators being from different sectors. In comparing the heterogeneity of types of organizations SHDs collaborated with, there was a statistically significant difference in group mean heterogeneity for collaboration between programme areas as determined by one-way ANOVA (*F* = 9.23, *p* < 0.001). Tukey’s post hoc test suggests that the obesity (mean difference + 0.11) and tobacco (mean difference + 0.07) programme areas had significantly higher group mean collaborator heterogeneity scores than the other chronic disease programme areas.

## Discussion

This study provides a look at the types of organizations that SHD health promotion/chronic disease units collaborate with, the extent to which these collaborating organizations belong to the health versus other sectors, and whether collaborators differ by chronic disease programme area. Our results suggest that for several major chronic disease programme areas, the types of organizations SHDs collaborate with remain heavily skewed towards the health sector. For a few select programme areas (obesity and tobacco), more frequent partnerships appear to be present for sectors outside of health.

In several areas, the results of this study are congruent with findings from the most recent ASTHO national survey on cross-sector collaborations among state health agencies. Although the ASTHO survey did not look at collaboration by programme area, they did report generally strong collaboration patterns between SHDs and organizations in the health sector [19]. For most agency types in the health sector, at least 90% of SHDs reported at least exchanging information with six of nine types of health sector agencies (hospitals, physician practices/medical groups, community health centres, health insurers, and emergency responders) [19].

The lower degree of collaborator heterogeneity among certain chronic disease areas can potentially be attributed to key factors associated with funding from government sources for activities and interventions within these programme areas. For example, in the cancer programme area, a larger proportion of funding may go towards screening-related activities that involve a clinical service, thus skewing collaborations in this programme area more towards organizations in the health sector [54]. Despite this, more multisector collaboration could still be encouraged, particularly in the areas of prevention and community-based living for survivors after treatment. While having many of the same mechanisms of comorbidity, diabetes, and cardiovascular disease as programme areas had significantly lower heterogeneity of collaborators compared to obesity. Again, although diabetes and cardiovascular disease may be more narrowly defined conditions involving specific clinical services related to diagnoses, treatment, and management compared to general obesity, these programme areas could also benefit from increased multisector collaborations. For example, lack of transportation is a commonly cited barrier to preventive screenings and clinical care for chronic disease management, so collaborations with local transportation planners and the transportation industry could improve access to preventive care and management of diabetes and cardiovascular disease [55]. Multisector collaborations addressing one risk factor for chronic disease (e.g., physical activity) may additionally impact other programme areas due to overlapping risk factors (e.g., diet, obesity) [56]. Finally, it is noteworthy that multisector collaboration was not particularly high in the “multiple chronic diseases” programme area. Participants who reported working across multiple programme areas included epidemiologists and evaluators and other roles that may not collaborate outside the SHD. Programme managers and chronic disease unit leadership teams with supervisory responsibilities across multiple programme areas would not be the SHD employees to directly work with the state-wide tobacco-free coalition, for example, but it is likely they would be working with Medicaid and other state-wide entities to set up collaborative initiatives.

When drawing conclusions from the study results, several limitations should be considered. Importantly, our sampling method was not designed to purposively sample across all states in a complete or uniform manner. Additionally, the decision to aggregate data from multiple respondents for SHD programme areas into a single case may provide a clearer broad overview of the collaboration patterns for each SHD, but some granularity in the data may be lost. Importantly, we only asked SHD participants who they collaborate with, so findings are unidirectional. We also did not assess the strength of partnerships, through for example asking about frequency or quality of collaboration for each kind of activity. Self-report carries the limitation of possible social desirability bias, which could inflate within-health-sector reporting (e.g., the Centers for Disease Control and Prevention [CDC] sometimes urges SHDs to collaborate with healthcare systems).

## Conclusions

Despite limitations, our study results provide important implications for SHD multisector collaborations moving forward, particularly taking into account major issues facing the public health system today. This is especially relevant in the context of health equity, because cancer, cardiovascular disease, and diabetes are among some of the chronic diseases with the largest differences in disease burden among population groups [57, 58]. Yet in these areas, SHD collaborations remained heavily skewed to health sector organizations, which are equipped primarily to offer a narrow range of specific clinical services and not address multiple community and social needs, a broad array of policies, and other critical social determinants of health [1, 59]. While services like health screenings, primary care, and hospitalization are important and readily provided by the health sector, other services such as transportation, education, housing, and income assistance linked strongly with social determinants of health are not adequately addressed by health sector organizations alone. Evidence suggests that equity and community health are enhanced when diverse organizational types can work hand in hand in tandem with the health sector [2]. Such evidence can inform how resources are directed to multisector collaborations. Compared to hospitals or clinics (to which access is systemically reduced for many minorities and vulnerable populations), municipal, faith-based, social, and educational institutions and organizations are far more influential and accessible community-based settings through which chronic disease prevention efforts may be channelled [2].

Although our study focuses primarily on health department collaborations in the context of chronic disease, the return of infectious disease to the forefront of consciousness in public health due to COVID-19 has highlighted more than ever the importance of strong multisector collaborations for all areas in population health [60–62]. In managing a coordinated public health response to the pandemic, SHDs and local public health agencies should collaborate with multiple sectors, including state education agencies, school districts, restaurant associations and chains, entertainment venues, and public transportation, to address issues such as social distancing protocols and reopening procedures [63]. Thus, in addition to chronic disease, issues in the areas of health equity and infectious disease increasingly highlight the importance of multisector collaborations for public health departments.

Despite evidence that over the last decade, SHDs are overall collaborating more and more with organizations outside of the health sector, our findings that several major chronic disease programme areas still collaborate largely with health sector organizations pave the way for several future research directions [12]. A direct next step is to further investigate mechanisms that facilitate collaboration of public health department work units with community-based organizations outside of the health sector. One potential approach to promoting multisector collaboration is specifically through key funding agency and stakeholder partnerships. For example, the CDC Action Communities for the Health, Innovation, and EnVironmental Change **(**ACHIEVE) initiative brought together national organizations from different sectors (e.g., YMCA-USA, National Recreation and Parks Association, National Association of County and City Health Officials) to create a cross-agency collaborative grant-funding structure for local community initiatives [1]. Effective partnerships are bidirectional and involve reciprocal investment from both parties; therefore, future research could study collaboration and the costs of collaboration from the vantage of multisector partnering organizations rather than largely from the perspective of health organizations such as public health departments. Finally, we recommend further investigation of effective collaboration processes between state-level and local-level multisector organizations, as organizations at the local level are often in the best position to promote health in neighbourhoods and communities [64, 65].

## Supplementary Information

**Additional file 1:** Frequency of state health department collaborations across all institution types.

## Data Availability

For related research materials, please see the Prevention Research Center in St. Louis, Washington University in St. Louis website, https://prcstl.wustl.edu/. Materials are available upon reasonable request.

## Abbreviations

ASTHO: Association of State and Territorial Health Officials
FQHCs: Federally Qualified Health Center
LHD: Local health department
NACDD: National Association of Chronic Disease Directors
SHD: State health department
