# Supplementary figures and images for: The power of partnerships: state public health department multisector collaborations in major chronic disease programme areas in the United States
Source: Health Res Policy Syst. 2022 Jul 8;20:80. doi: 10.1186/s12961-021-00765-3 (PMC9264297; doi:10.1186/s12961-021-00765-3)

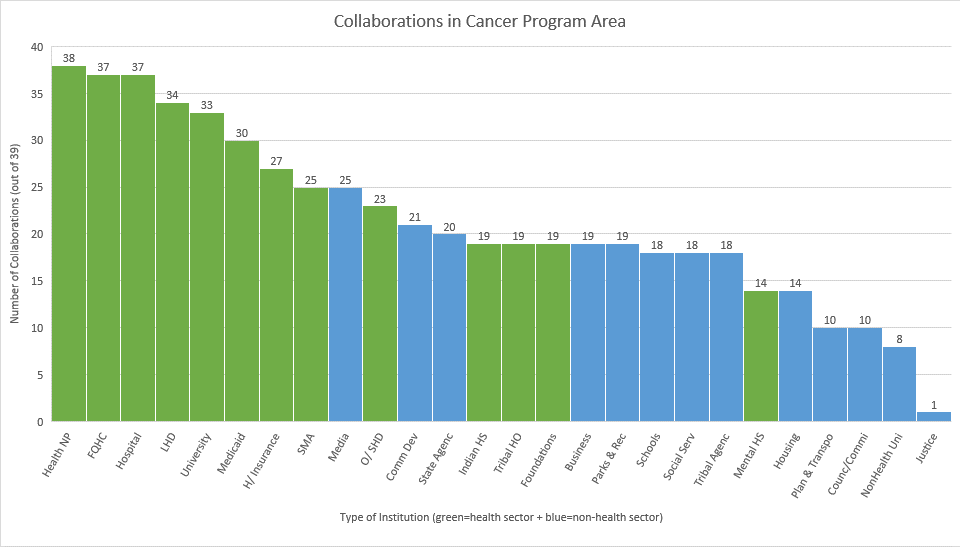


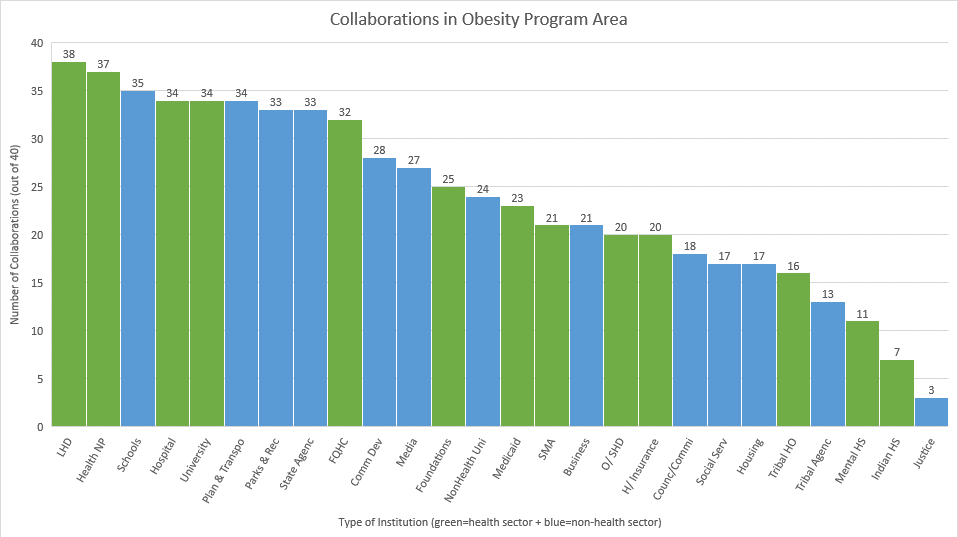


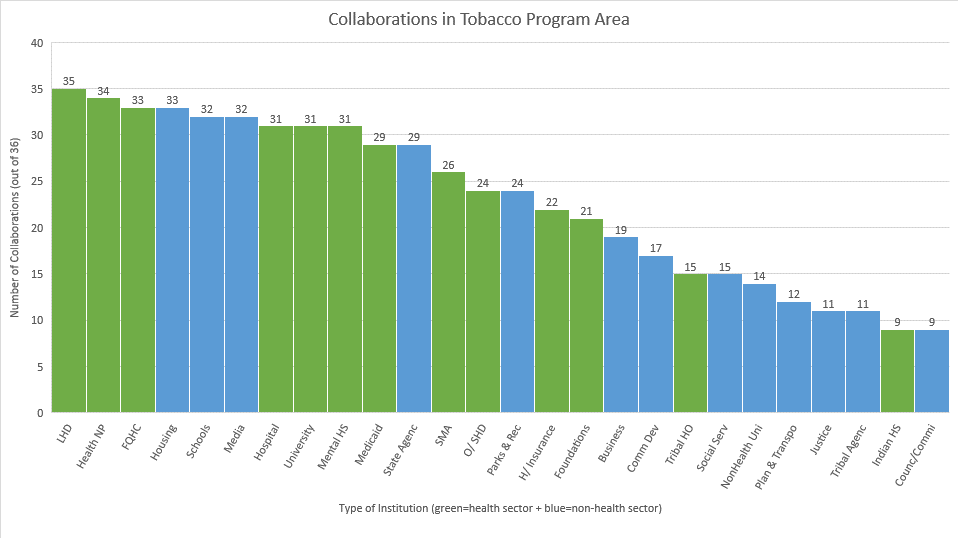


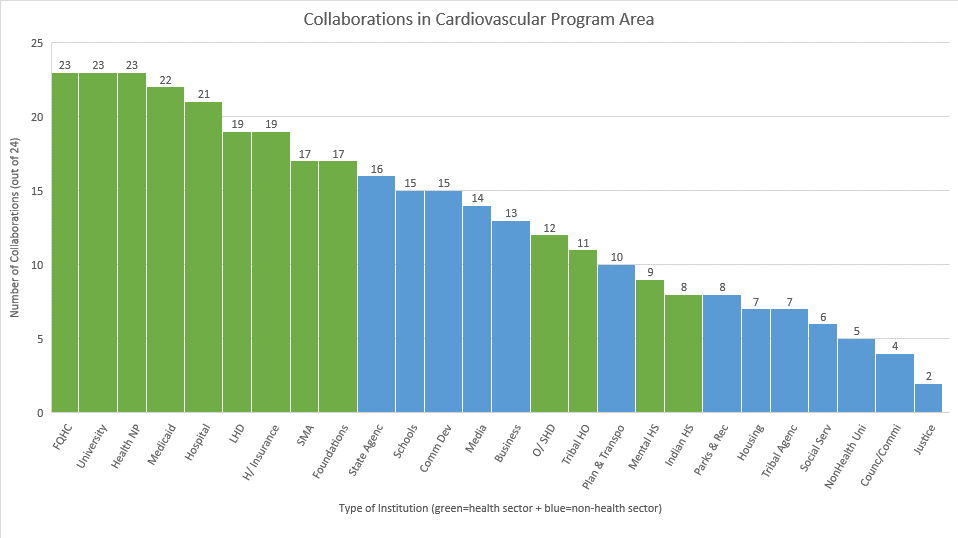


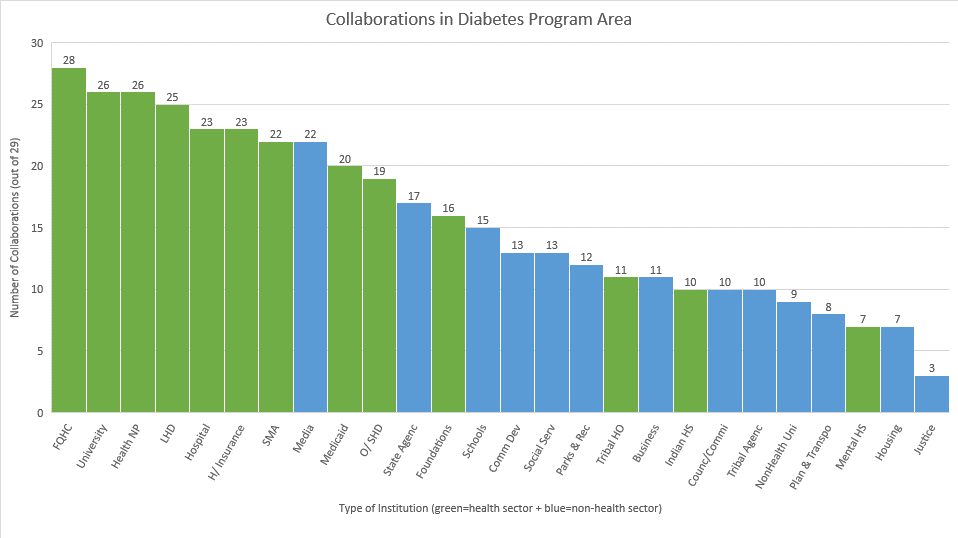


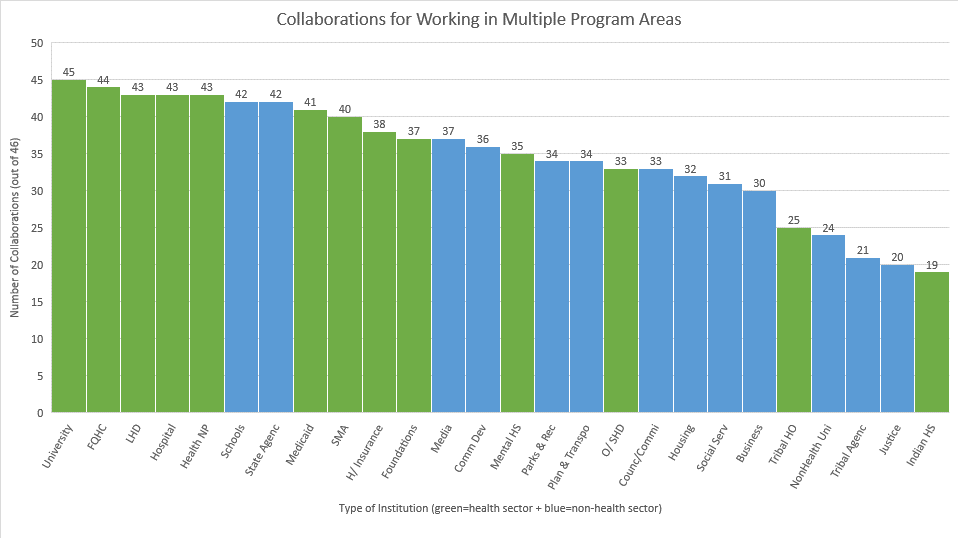

Supplement: Supplementary file 1 — Additional file 1: Frequency of state health department collaborations across all institution types. [file 12961_2021_765_MOESM1_ESM.docx]
